# Characterizing emergency departments to improve understanding of emergency care systems

**DOI:** 10.1186/1865-1380-4-42

**Published:** 2011-07-14

**Authors:** Anne P Steptoe, Blanka Corel, Ashley F Sullivan, Carlos A Camargo

**Affiliations:** 1 Department of Emergency Medicine, Massachusetts General Hospital, 326 Cambridge St, Suite 410, Boston, MA 02114 USA

## Abstract

International emergency medicine aims to understand different systems of emergency care across the globe. To date, however, international emergency medicine lacks common descriptors that can encompass the wide variety of emergency care systems in different countries. The frequent use of general, system-wide indicators (e.g. the status of emergency medicine as a medical specialty or the presence of emergency medicine training programs) does not account for the diverse methods that contribute to the delivery of emergency care both within and between countries. Such indicators suggest that a uniform approach to the development and structure of emergency care is both feasible and desirable. One solution to this complex problem is to shift the focus of international studies away from system-wide characteristics of emergency care. We propose such an alternative methodology, in which studies would examine emergency department-specific characteristics to inventory the various methods by which emergency care is delivered. Such characteristics include: emergency department location, layout, time period open to patients, and patient type served. There are many more ways to describe emergency departments, but these characteristics are particularly suited to describe with common terms a wide range of sites. When combined, these four characteristics give a concise but detailed picture of how emergency care is delivered at a specific emergency department. This approach embraces the diversity of emergency care as well as the variety of individual emergency departments that deliver it, while still allowing for the aggregation of broad similarities that might help characterize a system of emergency care.

## Introduction

The task of characterizing different emergency departments (EDs) is complicated by the fact that a wide array of entities function as EDs. This is particularly the case when studying EDs in different countries; yet, as the ACEP Section on International Emergency Medicine (EM) has emphasized, increased globalization trends both facilitate and require the exchange of knowledge and ideas within the international EM community in order to benefit global public health and health policy [1]. Since 2002, the Emergency Medicine Network (EMNet) has made such an effort by collecting information about emergency care in countries around the world as part of the National ED Inventories (NEDI) project. Countries studied, to date, include the United States (US, including more detailed work in 9 states), China (Beijing), Denmark, Nigeria (Abuja), Paraguay (Asuncion), Singapore, and Slovenia. (Much of the data provided in this paper comes from projects summarized on the NEDI website: http://www.emnet-nedi.org.). In conducting these studies, we expected to find international diversity among EDs [2,3]. We were surprised, however, by the ED diversity even within the US [4,5].

By repeating NEDI studies in multiple countries, it became clear that one can learn a great deal about a system of emergency care by examining its constituent EDs. Though this is not the only means of understanding systems of emergency care and emergency care can exist without EDs, examining emergency care systems via EDs yields a particularly rich portrait of local emergency care delivery. For instance, during the NEDI-Slovenia project, we found an unusual amount of variation in ED visit volume across the country. Upon examining the layout of EDs, we discovered that many Slovenian EDs are located within other specialty units and, therefore, may exist in multiple areas of a hospital. As such, complete visit volume data reflecting all emergency visits were not always obtainable, resulting in the observed inconsistency. This example points to the need to establish common ways of understanding international EDs before we can hope to understand even basic data on a national emergency care system. Furthermore, developing common terms for characterizing EDs is a necessary first step if we wish to categorize EDs by capabilities or other measures. As Arnold and Holliman point out, previous attempts to categorize emergency care systems internationally have experienced problems of oversimplification [6,7]. Rather than relying on regional or national characteristics to encompass local variation, observing systems of emergency care through individual EDs meets the problem by employing the opposite approach, aggregating local data to characterize regional emergency care. In this paper, we aim to outline a methodology for studying emergency care internationally by examining ED characteristics. Such a methodology is replicable across a wide variety of emergency care systems, and provides a wealth of information that can inform future research and public health efforts in a particular country.

Characterizing EDs requires first defining what is meant by the term "ED." Even prominent EM organizations, such as the American College of Emergency Physicians, do not offer a clear-cut definition, a situation that may reflect the complexity of pinpointing one (Table 1). Though it is difficult to incorporate every service providing emergency care into one compact definition, we have created primary and secondary criteria that describe all the ED facilities that we include in NEDI studies. We believe that the primary criterion for being considered an ED is the provision of immediate, often stabilizing, care for patients with emergent medical needs. However, this criterion alone cannot distinguish the ED from other acute medical services. We believe that the secondary criterion for defining an ED is that it provides a base level of availability and accessibility. Usually, this means that the ED provides emergency care "round-the-clock," (24 hours per day, 7 days per week, 365 days per year) with no restriction on who can access that care.

**Table 1:** Different definitions of an emergency department

Perspective	Definition	Source
Academic	"The worldwide definition... traditionally implies the *rapid and appropriate care of victims of traumatic and medical emergencies*"	Sikka and Margolis [14]
National organization	"An organized hospital facility for the provision of *unscheduled outpatient services to patients whose conditions are considered to require immediate care*"	American Hospital Association [18]
	"A hospital *facility for the provision of unscheduled outpatient services to patients whose conditions require immediate care *and is staffed 24 hours a day. If an ED provided emergency services in different areas of the hospital, then all of these emergency service areas are [included]... Off-site EDs that are open less than 24 hours are included if staffed by the hospital's ED"	Burt and McCaig (The Center for Disease Control and Prevention) [19]
National government	A facility that "is publicized to the public by name, posted signs, advertising or other means as a place that *provides care for emergency medical conditions on an urgent basis *without requiring a previously scheduled appointment," or "a department that is designated as an emergency department by state licensure" or "a department that, during the prior calendar year, provided at least one-third of all its outpatient visits for the *treatment of medical conditions on an urgent basis *without requiring a previously scheduled appointment... Labor and Delivery Departments and Urgent Care Centers are considered to meet the above criteria. This definition applies whether the department is on or off campus, as long as it is a department of the hospital or critical access facility"	The Emergency Medical Treatment and Active Labor Act [10]
State government	"A hospital department consisting of staff, facilities, and resources to *provide emergency medical care *for large numbers of emergency patients"	New York State Public Health Law [20]
Hospital	We provide state-of-the-art evaluation and *treatment for patients with a full spectrum of emergency medical needs*"	New-York Presbyterian Hospital, New York City [21]
Patient	"A place to go *when you need to be seen by a doctor quickly *24/7. They take care of everything and everyone there"	Anonymous patient, St. Luke's Roosevelt Hospital, New York, New York.
	"The place people go when they feel their medical problem is serious enough that *they can't wait to be seen by their regular doctor*. The ED is always open, and will care for any person's medical problem regardless of their ability to pay"	Anonymous patient, Massachusetts General Hospital, Boston, Massachusetts

Abbreviation: ED, emergency department

Even when applying both primary and secondary criteria, one can find exceptions to this ED definition. In these cases, it helps to consider whether, in a given emergency care system, that type of ED represents a significant component of emergency care for patients in that region or nation. Often, exceptions to the ED definition do not reflect the way in which most people receive emergency care, complicating a regional or national portrait of emergency care without contributing enough information about overall routes of emergency care to merit inclusion. We have encountered exceptions to the primary and secondary criteria in many countries. For example, some US federal EDs are also available to members of the general population. Others are designed for use by a specific group, like Indian Heath Service hospital EDs, or may have reduced accessibility because of their secure location, like military hospital EDs. Such EDs might not be included in an examination of national emergency care systems because, although they may serve some members of the civilian population, they likely do not provide an emergency care route for the total, general population in their region. Similarly, medical facilities at both public institutions (e.g. prison hospitals) and private institutions (e.g. college infirmaries) will occasionally have their own ED. Rarely are institutional EDs easily accessed or frequently utilized by the general public, so they are usually excluded from studies of emergency care systems. A particularly challenging exception to the secondary ED criterion is provided by insurance-linked EDs. Such EDs provide care for patients through a certain insurance plan, though we have found that most would at least stabilize any patient. In Asuncion, Paraguay, we judged these EDs to provide a major route of emergency care for the total population and included them in our NEDI study. Yet another unique permutation of the ED definition is the medical specialty ED. Such EDs may or may not meet the primary criterion of being an ED, depending upon whether they only provide emergency care for their specialty or provide treatment for most emergency medical needs (i.e., are "full-service" EDs). For instance, in the US, some cardiac and psychiatric hospitals have general EDs, but others provide emergency care only in their specialty. The latter facilities, though they provide emergency care, would not be considered an ED for the purposes of understanding an emergency care system. The myriad of examples provided demonstrates that routine exceptions to ED availability and accessibility exist and should be considered carefully for inclusion in an analysis of an emergency care system. This is particularly true when assessing emergency care outside the developed world [8]. It is also possible that emergency care may exist in countries lacking an emergency care system. Including the secondary criterion provides one way to distinguish between the presence of emergency care and an emergency care system.

### Four ways to characterize EDs

Although the diversity among the EDs included in the NEDI studies based on the primary and secondary criteria was staggering, certain basic characteristics proved useful for describing EDs in seven countries across five continents. When viewing EDs from the perspective of how patient care is delivered, we identified four main characteristics that one can use to describe EDs: (1) physical location, (2) physical layout, (3) time period open to patients, and (4) patient type served. Considering each of these characteristics can, in turn, yield many different varieties of individual EDs (Table 2). There are many more ways to describe EDs. However, we have found that these four characteristics are particularly well suited to describing a wide variety of care contexts. That is, they represent a basic common framework to which other factors can be added. For example, the distinction between rural and urban settings is an important descriptor of US emergency care; yet the terms have less value in countries where an emergency care system does not yet exist in rural areas. To then attempt to compare countries using rurality is fraught with difficulty. Gathering information about the four ED characteristics allows us to collect data about the scope and practice of emergency care delivery at specific sites. Aggregating such data from several EDs provides one means of assessing the landscape of emergency care in a regional or national system and common terms by which to compare these systems across countries.

**Table 2:** Four categories of emergency department Characteristics

**1. ED Location**
a. Hospital-based
b. Freestanding (non-hospital-based)
i. Satellite
ii. Autonomous
iii. Primary-care-based

**2. ED Layout**
a. Contiguous
i. With triage to service
ii. Without triage to service
b. Non-contiguous

**3. Time period open to patients**
a. Full-time
b. Part-time
c. Seasonal
d. Alternating

**4. Patient type served**
a. General population
i. Combined
ii. Separate
b. Adult
c. Pediatric

Abbreviation: ED, emergency department

#### 1. Physical location of EDs

One of the most basic features of emergency care is where that care is provided. Characterizing EDs by location produces two main groups: hospital-based EDs and freestanding EDs. Hospital-based EDs are typically located in a general acute care hospital, but may also be found in specialty hospitals (Table 3). A second group of EDs encompasses all EDs not based within a hospital, or so-called "freestanding" EDs. Freestanding EDs can be further characterized as satellite EDs, autonomous EDs, and primary-care-based EDs. Satellite facilities have an official affiliation with a particular hospital, while autonomous facilities do not (Table 3). In primary-care-based EDs, as their name suggests, emergency service is incorporated into primary care, as is the case with primary care practices or mother and child clinics in some countries. In such EDs, primary care physicians provide 24/7 general emergency care in addition to regular primary care (Table 3).

**Table 3:** Recent examples of emergency departments by major characteristics^1^

*ED characteristic *	*Group*	***US example***^2^	*International example*
Physical location	Hospital-based ED	New York-Presbyterian Weill Cornell Medical Center, New York, NY	Tan Tock Seng Hospital, Singapore
	Satellite ED	INOVA Health System's four Emergency Care Centers, northern VA	
	Autonomous ED	Texas Emergency Care Center, Pearland, TX	
	Primary care-based ED		Health Care Center Jesenice, Jesenice, Slovenia
Physical layout	Contiguous ED without triage to service	The Cleveland Clinic, Cleveland, OH	Centro Médico La Costa, Asuncion, Paraguay
	Contiguous ED with triage to service		Bispebjerg Hospital, Copenhagen, Denmark
	Non-contiguous ED		University Center Maribor, Maribor Slovenia *(medical and surgical emergencies are handled in separate buildings, and other specialties have separate emergency areas)*
Time period open to patients	Full-time ED	Ronald Reagan UCLA Medical Center, Los Angeles, CA	Kings Care Hospital, Abuja, Nigeria
	Part-time ED		Cami Altamira, Bogota, Columbia
	Seasonal ED	Millville Emergency Center, Millville, DE *(a 24/7 ED only from Memorial Day to Labor Day)*	
	Alternating ED		Centre Hospitalier Emile Mayrisch Esch/Alzette Esch-sur-Alzaette, Luxembourg and the Centre Hospitalier de Luxembourg Clinique d'Eich, Luxembourg, Luxembourg
Patient type served	Combined general population ED	The Mayo Clinic, St. Marys Hospital, Rochester, MN	Number Six Hospital, Beijing, China
	Separate general population ED	Kapi'olani Medical Center for Women and Children, Honolulu, HI	National University Hospital, Singapore
	Adult ED	Holy Cross Hospital Seniors' Emergency Center, Silver Spring, MD	Tan Tock Seng Hospital, Singapore
	Pediatric ED	The Children's Hospital, Aurora, CO	Kandang Kerbou Hospital, Singapore

Abbreviation: ED, emergency department

1. The status of hospitals is constantly shifting. Most data in this table were gathered with reference to 2007, though US-based examples were confirmed in late 2009 and early 2010.

#### 2. Physical layout of EDs

Emergency care may also be provided in several different layouts within a facility. Characterizing EDs by physical layout distinguishes the many ways that EDs are designed and yields two main groups: contiguous and non-contiguous. In a contiguous ED, medical and surgical emergencies are treated in one or adjacent areas. Contiguous EDs can be further described as having or lacking triage to service. "Triage to service" does not refer to the process of patients being admitted to the hospital from the ED, but rather to the process whereby patients arriving at the ED are directed to emergency care from non-EM specialties (e.g., to a medical or surgical team; Table 3). A contiguous ED with triage to service is often staffed by physicians from many different specialties (e.g., surgeons, internists) who are employed by their respective departments and who treat emergencies related to their field. In contrast, a contiguous ED without triage to service is often staffed by physicians who provide emergency care to all patients (Table 3). We recognize that pre-hospital care is often an important component of triage to service, but the marked heterogeneity of pre-hospital care is beyond the scope of this article.

A patient seeking emergency care may not always be seen in a unified, or contiguous, area, but rather in one of several locations, depending on their particular need. For instance, a patient with a broken ankle might receive care in the Orthopedics Department, while a patient presenting at the same facility with a myocardial infarction would be seen in the Cardiology Department. This ED layout might be called a non-contiguous design. Even in a non-contiguous ED, a central triage location usually helps direct patients to the proper non-EM emergency area, though patients also can be triaged in the pre-hospital setting (Table 3).

#### 3. Time period open to patients of EDs

EDs may also be characterized according to when they provide emergency care. Although the secondary criterion of an ED is that it provides a base level of availability, EDs may sometimes provide care that is less than round-the-clock because of the limitations or special needs of a particular location. If characterized in this way, EDs tend to fall into four groups: full-time, part-time, seasonal or alternating. A full-time ED provides care 24 h per day, 7 days per week, 365 days per year (Table 3). In contrast, a part-time ED is open less than 24 h per day, 7 days per week, 365 days per year. In some countries, part-time EDs can represent a major vehicle of emergency care, though they usually are open at least 150 of 168 hours per week and 365 days per year (Table 3). The existence of part-time EDs raises the issue of "urgent care centers" in the US [9]. These centers are typically open less than 150 h per week, are limited in the scope of service they can provide, and do not represent a major way that individuals access emergency care. For this reason, though they play a supplemental role in overall emergency care, we did not include them in the NEDI-USA database [4,5]. Similar reasoning may be applied to seasonal EDs, which are only open during one portion of the year. Seasonal EDs may be either full-time or part-time while they are open, and are generally found in areas whose population varies by season, such as beach and ski resort areas (Table 3). If seasonal EDs represent an important component of care for a population that is itself seasonal, they should be included in studies of emergency care systems. Finally, alternating EDs are those which share responsibility for providing 24/7 emergency care to a population. Though each hospital may have an "ED" that, when considered alone, may not qualify as such due to its restricted hours of availability, both hospitals together are able to provide full-time emergency coverage through their alternating ED system. Although such EDs are not common in the US, they are an element of emergency care in rural areas of other countries (Table 3).

#### 4. Patient type served by EDs

Characteristics of patients themselves are an important part of describing an ED. Although the secondary criterion of an ED stipulates that a facility is generally accessible to the public, we have encountered many more nuanced variations on accessibility based on local emergency care needs. When characterizing EDs by the type of patient served, three main groups appear: general population EDs, adult EDs, and pediatric EDs. General population EDs serve all patients regardless of age, sex, race/ethnicity, or other major sociodemographic factors. General population EDs may be further characterized as combined or separate. Combined general population EDs provide care for all patients in one area, while separate general population EDs provide care to different groups of patients in distinct areas within one facility depending upon specific patient characteristics. The most common population characteristic that distinguishes these two types of general population EDs is age, as demonstrated by children and adults being seen in separate locations within a facility (Table 3). However, not all EDs primarily serve both children and adults. Adult EDs primarily serve adults, even if - at least in the US - they are technically accessible to individuals of all ages under the Emergency Medical Treatment and Active Labor Act [10]. Geriatric EDs, designed for patients over 65 years of age, represent one particular subset of adult EDs (Table 3). In contrast, pediatric EDs primarily serve children, though they routinely encounter the occasional adult patient (Table 3) [11]. The definitions of an adult and pediatric EDs are complicated by the many different definitions of "adult" and "pediatric." For example, the East Georgia Regional Medical Center in Georgia (Statesboro, GA) places the cutoff between pediatric and adult patients at 12 years old, but it is 18 years old at the Oregon Health and Science University Hospital (Portland, OR) and 21 years old at Children's Hospital Boston (Boston, MA).

### Using ED characterization methods to understand complex situations

**Figure 1:**
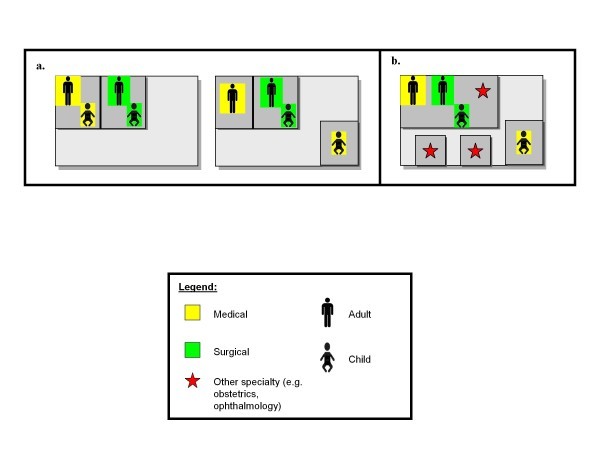
**Examples of combining characteristics to describe individual emergency departments**. **a **The schematic on the *left *depicts a contiguous ED with triage to service. The schematic on the *right *depicts a non-contiguous ED. Other specialty care is not depicted to emphasize the difference in layout between these two similar ED types. The difference between the two categories of ED hinges on the location of pediatric medical care. In the non-contiguous ED, this care is located in a different place within the healthcare facility than the remainder of emergency care. **b **This schematic depicts an ED with adult medical and surgical care, as well as pediatric surgical care and some specialty care, in one location, but pediatric medical care and specialty care in separate locations within a healthcare facility.

Only in combining multiple ED characteristics does the overall patient experience become apparent. Some combinations appear more frequently than others, but there are as many as 120 different ways in which ED characteristics may combine, encompassing a wide variety of EDs (Table 4). Using multiple ED characteristics simultaneously can capture efficiently ED designs that would be considered quite unusual to a US audience. For instance, the University Medical Centre Ljubljana in Slovenia provides care through a non-contiguous ED that has both separate and combined general population care (Figure 1). In this hospital, other specialty emergencies are treated in a different location than medical, surgical, or OB-GYN emergencies, making the ED non-contiguous. Furthermore, pediatric and adult emergency medical care is provided in different locations within the hospital, a separate general population model, but both adult and pediatric surgical emergencies are handled in the same location, a combined population model.

**Table 4:** Selected combinations of emergency department categories

Characteristics	Example
1. Contiguous ED without triage to service and with separate general population care	Massachusetts General Hospital, Boston, MA, USA
2. Contiguous ED without triage to service and with combined general population care	Sanatorio Italiano, Asuncion, Paraguay
3. Contiguous ED with triage to service and with combined general population care	Number Six Hospital, Beijing, China
4. Contiguous ED with triage to service, combined general population surgical care, and separate general population medical care	Regionshospitalet Holstebro, Holstebro, Denmark
5. Non-contiguous ED with combined general population care	Køge Sygehus, Køge, Denmark
6. Non-contiguous ED with separate general population medical care and combined general population surgical care	University Medical Center Maribor, Maribor, Slovenia
7. Contiguous pediatric ED without triage to service	The Children's Hospital, Aurora, CO, USA
8. Contiguous pediatric ED with triage to service	Instituto Privado de Nino, Asuncion, Paraguay
9. Contiguous, adult ED without triage to service	Beth Israel Deaconess Medical Center, Boston, MA, USA
10. Non-contiguous, adult ED	Nykøbing F. Sygehus, Nykøbing Falster, Denmark

Abbreviation: ED, emergency department

### Categorizing emergency departments by capabilities

The issue of categorizing EDs by their emergency care capabilities is not directly addressed by this paper. We consider it crucial that the task of *characterizing *EDs be distinguished from that of *categorizing *EDs. For this reason, we have avoided many ED and emergency care descriptors that we consider categorization, not characterization, tools. These may include: number of beds, ED complaints managed, type of providers used, and special capacity designations, such as those of trauma center or stroke center in the US [12]. Only after the basic landscape of emergency care has been described can one effectively begin to categorize EDs. That is, characterizing EDs provides a basic framework of understanding that can be supplemented by ED categorization. However, the details of categorizing EDs by their emergency care capability are beyond the scope of this paper.

### Using ED characterization to understand emergency care systems

Because the ED represents a major facet of emergency care, it can provide a valuable method of describing emergency care systems. The benefits of this approach are two-fold: it allows for diversity and provides a neutral ground upon which to compare emergency care systems. This may be a particularly useful starting point in international EM, because previous models for assessing emergency care systems in foreign countries have received criticisms of oversimplification and implied categorization. For instance, the "geographic" model reduces the many different systems of emergency care to just two: the "Anglo-American," or hospital-based emergency care, and the "Franco-German," or pre-hospital-based emergency care [6,13]. Other emergency care systems are characterized in terms of how they follow or deviate from these two systems--an approach that has provoked controversy [6,7]. Another model, which might be termed the "progress" model, places emergency care systems into three groups: underdeveloped, developing, and mature. Each group measures certain systemic indicators of EM, including its status as a specialty, the amount of training that providers have had, the presence of a pre-hospital system, and the sophistication of patient care and management systems [7]. Though the Progress Model increases the number of groups in which EDs may be placed and the number of factors that contribute to placing an ED in each, it still limits the ways that EDs and emergency care systems can be described to just three and implies an inherent categorization favoring the "mature" system.

In recent years, international EM experts have called repeatedly for a more nuanced way of describing emergency care systems [6,14]. Such attempts have been made, but none has yet been widely adopted; and each has still focused on system-wide measures [6,15-17]. For instance, one of the only multinational studies of emergency care systems to date, though it also looked at some ED features, focused on whether EM had a specialty status by looking at whether physicians could receive medical education, residency training, fellowship, and board certification in EM, and whether a national EM organization, research field, journal, or database existed [16]. While these are laudatory achievements, assessing emergency care on the level of the ED provides one way to meet international EM researchers' call for a more nuanced system while facilitating the aggregation of ED features to understand a larger landscape of emergency care. It also reveals just how much variation exists between and within countries. Similar local contexts may produce similar, even virtually identical, systems of emergency care, but this need not be a requirement for effective care. Similarly, in many places, imitating U.S. emergency care may be neither immediately feasible nor necessary. Viewing emergency care on the ED level allows researchers to track the development of an emergency care system while embracing the fact that systems of emergency care must adapt to local circumstances to succeed.

## Summary

Using general, system-wide indicators to characterize systems of emergency care may render an oversimplified portrait of regional or national emergency care that researchers have previously identified as problematic [6,7]. Defining and comparing individual EDs may help assess regional or national emergency care systems without losing a sense of local variation in emergency care delivery. Although many different ED types can combine to create an emergency care system, looking at ED-specific characteristics still allows for comparison of different systems, while incorporating the idea that local circumstances may require local solutions. An ED-centric approach to assessing emergency care is, however, only one of several ways to frame a discussion of international emergency care. The major advantage of focusing on ED characteristics is that it provides detailed but comparable information about actual emergency care delivery on the local level. This understanding can form a foundation upon which categorization methods can then build.

As demonstrated by numerous examples in this article, there are many more ways to structure an ED than the traditional hospital-based model that most in the US would understand as an "ED." Using key ED characteristics to capture this diversity may provide a better approach for analyzing emergency care systems across the globe. Our approach is quantifiable, allows tracking over time, and cross-country comparison - while paying attention to the local context out of which ED designs are born. It is also important to remember that our model is flexible. With each new study, we have found ourselves expanding upon the basic elements presented here while continuing to operate within the same general methodological framework. The core data from the four basic ED characteristics have served as a way for us to understand and compare emergency care systems before we are able to assess a new emergency care system. In future years, EM researchers may perform outcome studies to examine the clinical and economic effectiveness of different ED types in managing the broad array of conditions that present for emergency care.

## Author information

Ms. Steptoe is a graduate of Harvard College and a former research fellow at the Emergency Medicine Network (EMNet, http://www.emnet-usa.org) at Massachusetts General Hospital. Dr. Corel is a graduate of the University of Ljubljana in Slovenia. She is an internist and emergency physician, and a former research fellow at EMNet. Ms. Sullivan is a graduate of Bowdoin College and Tufts University. She is a biostatistician/epidemiologist at Massachusetts General Hospital, as well as Associate Director of EMNet. Dr. Camargo is an emergency physician at the Massachusetts General Hospital; and Associate Professor of Medicine & Epidemiology at Harvard Medical School. He holds degrees from Stanford University, University of California Berkeley, University of California San Francisco, and Harvard University. Dr Camargo is founder and ongoing Director of EMNet.

## Competing interests

The authors declare that they have no competing interests.

NEDI-International Country Coordinators (to date):

Venkataraman Anantharaman MBBS, FRCP (Singapore General Hospital, Singapore); Philip Anderson, MD (Beth Israel Deaconness Medical Center, Boston, USA); Juan A Caceres, MD (Ministry of Public Health, Asuncion, Paraguay); Blanka Corel, MD (Massachusetts General Hospital, Boston, USA); Itsabo Oshiomogho, MBBS, MS (Brandeis University, Waltham, MA, USA); Soren Stagelund, MD (Hvidovre Hospital, Hvidovre, Denmark); and Jun Xu, MD (Peking Union Medical College, Beijing, China).

*NEDI-USA State Coordinators (to date)*:

Adit A. Ginde, MD, MPH (University of Colorado Denver School of Medicine, Aurora, CO); Jonna Graves, MD (Ivinson Memorial Hospital, Laramie, WY); Daniel A. Handel, MD, MPH (Oregon Health and Science University Medical Center, Portland, OR); Talmage M. Holmes, PhD, MPH (University of Arkansas Medical School); Ray E. Keller, MD (Fletcher Allen Healthcare, Burlington, VT); Ali S. Raja, MD, MBA (Brigham and Women's Hospital, Boston, MA); John Rogers, MD (Monroe County Hospital, Forsyth, GA); and Daniel C. Smith, MD (Queens Medical Center, Honolulu, HI)

EMNet Steering Committee:

Carlos A. Camargo, Jr., MD, DrPH (Chair); Sunday Clark, MPH, ScD; Robert A. Lowe, MD, MPH; Jonathan M. Mansbach, MD; Ashley F. Sullivan, MPH, MS; and Scott T. Wilber, MD, MPH.

EMNet Coordinating Center:

Carlos A. Camargo, Jr., MD, DrPH (Director); Dinah Chen; Erica Eagan; Janice A. Espinola, MPH; Tate Forgey, MA; Natalie Mazur; Sara Mills; Ashley F. Sullivan, MS, MPH; Pornthep Tanpowpong, MD, MPH; and Sarah A. Ting, PhD.

## Authors' contributions

All authors contributed to the conception, development and preparation of this article.
